# Supplementary material for: Alanine aminotransferase controls seed dormancy in barley
Source: Nat Commun. 2016 May 18;7:11625. doi: 10.1038/ncomms11625 (PMC4873977; doi:10.1038/ncomms11625)
Supplement: Supplementary Information — Supplementary Figures 1 - 10 and Supplementary Tables 1 - 8 [file ncomms11625-s1.pdf]

```
                10         20         30         40         50         60
       ATGTCGTACAACAAGACCGCCTCCATCACCGCCGAGACCATAAACCCCAAGGTCAAGATC
    1  M  S  Y  N  K  T  A  S  I  T  A  E  T  I  N  P  K  V  K  I       20

                70         80         90        100        110        120
       TTCGACTACGAGCCCTGCGGGGAGATCGCCAGGCACGCAGAGCGGTTGGAGCAGGAGATG
   21  F  D  Y  E  P  C  G  E  I  A  R  H  A  E  R  L  E  Q  E  M       40

               130        140        150        160        170        180
       GAGAAGAGCCCAGGTTCTCGCCCTTTCCCAGAGATTATATACTGCAACCTTGGGAACCCT
   41  E  K  S  P  G  S  R  P  F  P  E  I  I  Y  C  N  L  G  N  P       60

               190        200        210        220        230        240
       CAGGCTCTCGGGCAGCGCCCGATAACCTTCTTTCGTGAGGTTATTTCTCTGTGTGACAAT
   61  Q  A  L  G  Q  R  P  I  T  F  F  R  E  V  I  S  L  C  D  N       80

               250        260        270        280        290        300
       CCAGCTCTCCTGCACAGAGATGAAACCCGTATGTTATTCAGTCCATGTGCCATAAATAGA
   81  P  A  L  L  H  R  D  E  T  R  M  L  F  S  P  C  A  I  N  R      100

               310        320        330        340        350        360
       GCGCGGAAGATTATCGAGTCGATGCCCGGAAGAAACACCGGTGCATATACCAACAGTCAG
  101  A  R  K  I  I  E  S  M  P  G  R  N  T  G  A  Y  T  N  S  Q      120

               370        380        390        400        410        420
       GGAATCCGAAGTTTGCGCGAAGCGGTTGCAAGTGGAATCGCTGCAAGAGATGGTTTTCCA
  121  G  I  R  S  L  R  E  A  V  A  S  G  I  A  A  R  D  G  F  P      140

               430        440        450        460        470        480
       TCAAGACCGGAAGACATCTTTCTGACGGATGGAGCGAGTTCAGCGATCAATTTGAGTATG
  141  S  R  P  E  D  I  F  L  T  D  G  A  S  S  A  I  N  L  S  M      160

               490        500        510        520        530        540
       CAGATACTCATTAGGTCCCAAGAAGATGGCATTTTATGCCCTTTACCCGAATATCCATTA
  161  Q  I  L  I  R  S  Q  E  D  G  I  L  C  P  L  P  E  Y  P  L      180

               550        560        570        580        590        600
       TACTCGGCGTCCATTATACTTCATGGTGGGACTATGGTACCGTACAATCTTAGCGAGGAT
  181  Y  S  A  S  I  I  L  H  G  G  T  M  V  P  Y  N  L  S  E  D      200

               610        620        630        640        650        660
       GGTGATTGGGGGCTTGAGATTTTCGAAGTAAAGAGGTGCTTgGAGGAGGCACGCATCGCA
  201  G  D  W  G  L  E  I  F  E  V  K  R  C  L  E  E  A  R  I  A      220

               670        680        690        700        710        720
       GGTTTGACTGTTCGTGCTATGGTGATCATAAATCCCGGAAACCCAACGGGACAGGTACTG
  221  G  L  T  V  R  A  M  V  I  I  N  P  G  N  P  T  G  Q  V  L      240

               730        740        750        760        770        780
       TCTGTCACCAACCAGGAGGAGATAGTAGAATTTTGTCGGAAAGAAGGCTTGGTTATCCTT
  241  S  V  T  N  Q  E  E  I  V  E  F  C  R  K  E  G  L  V  I  L      260

               790        800        810        820        830        840
       GCCGATGAGGTATACCAAGATAATGTCTATGTGGAGGATAGGAAGTTCAATTCTTTCAAG
  261  A  D  E  V  Y  Q  D  N  V  Y  V  E  D  R  K  F  N  S  F  K      280

               850        860        870        880        890        900
       AAAGTTGCCAGATCACTTGGGTgTGACGAGAATGACATCTCCATAGTGTCATTTCATTCG
  281  K  V  A  R  S  L  G  C  D  E  N  D  I  S  I  V  S  F  H  S      300

               910        920        930        940        950        960
       GTTTCGATGGGTTACTCTGGAGAATGTGGCAGAAGGGGAGGCTACATGGAGATATGTGGT
  301  V  S  M  G  Y  S  G  E  C  G  R  R  G  G  Y  M  E  I  C  G      320

               970        980        990       1000       1010       1020
       TTTGGAGATGATGTGATGGGTGAGATTCGCAAGGTGGCTTCTGTGACTCTTTGCCCCAAC
  321  F  G  D  D  V  M  G  E  I  R  K  V  A  S  V  T  L  C  P  N      340

              1030       1040       1050       1060       1070       1080
       ACAAATGGTCAAATTCTTACTAGCCTTGCGATGGATCCACCAAAGCTAGGAGATGGTTGT
  341  T  N  G  Q  I  L  T  S  L  A  M  D  P  P  K  L  G  D  G  C      360

              1090       1100       1110       1120       1130       1140
       TTCGAGGCTTTCATGGCTGAAAAGGAAGAAAcCAGTACATCTTTGGCCAAGCGCGCCAAG
  361  F  E  A  F  M  A  E  K  E  E  T  S  T  S  L  A  K  R  A  K      380

              1150       1160       1170       1180       1190       1200
       ACCTTGGTGAACGCATTCAGCAGCCTGGAGGGAATGACCTGCAACAAAGTAGAGGGCGCA
  381  T  L  V  N  A  F  S  S  L  E  G  M  T  C  N  K  V  E  G  A      400

              1210       1220       1230       1240       1250       1260
       ATCTACGCCTTCCCACGCATCCATCTCCCAGCAGCGGCGATCAAAGCCGCCAAGGCCGAG
  401  I  Y  A  F  P  R  I  H  L  P  A  A  A  I  K  A  A  K  A  E      420

              1270       1280       1290       1300       1310       1320
       GACaTGTCCCCAGACACGTTCTACGCGTGCCGTCTTCTCGATGCCACCGGAATCGCGGTC
  421  D  M  S  P  D  T  F  Y  A  C  R  L  L  D  A  T  G  I  A  V      440

              1330       1340       1350       1360       1370       1380
       GTCCCTGGCTCTGGATTCCACCAGATCTCTGGGCGCAACACGGCGACCGGGACATGGCAT
  441  V  P  G  S  G  F  H  Q  I  S  G  R  N  T  A  T  G  T  W  H      460

              1390       1400       1410       1420       1430       1440
       ATCcGGTGCACTATCCTTGTCGCCGAGGACAAGATCGAGGCAATGATCGCGCGCCTCAAG
  461  I  R  C  T  I  L  V  A  E  D  K  I  E  A  M  I  A  R  L  K      480

              1450       1460       1470       1480
       GCCTTCCACGAGTCCTTCATGAACGAGTTCCGCGACCGAAGCTGA
  481  A  F  H  E  S  F  M  N  E  F  R  D  R  S  *     495
```

Supplementary Fig. 1| Nucleotide and amino acid sequences of *qsd1* in dormant wild barley H602.

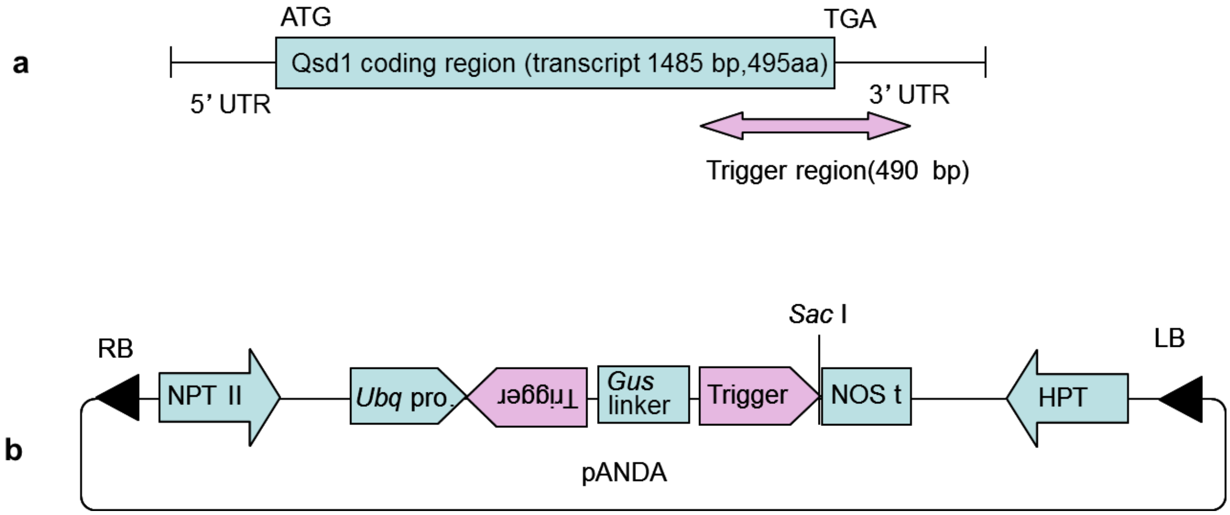

Supplementary Fig. 2| RNAi knockdown construct of *Qsd1*. a, Trigger region, **b**, RNAi trigger vector; pANDA.

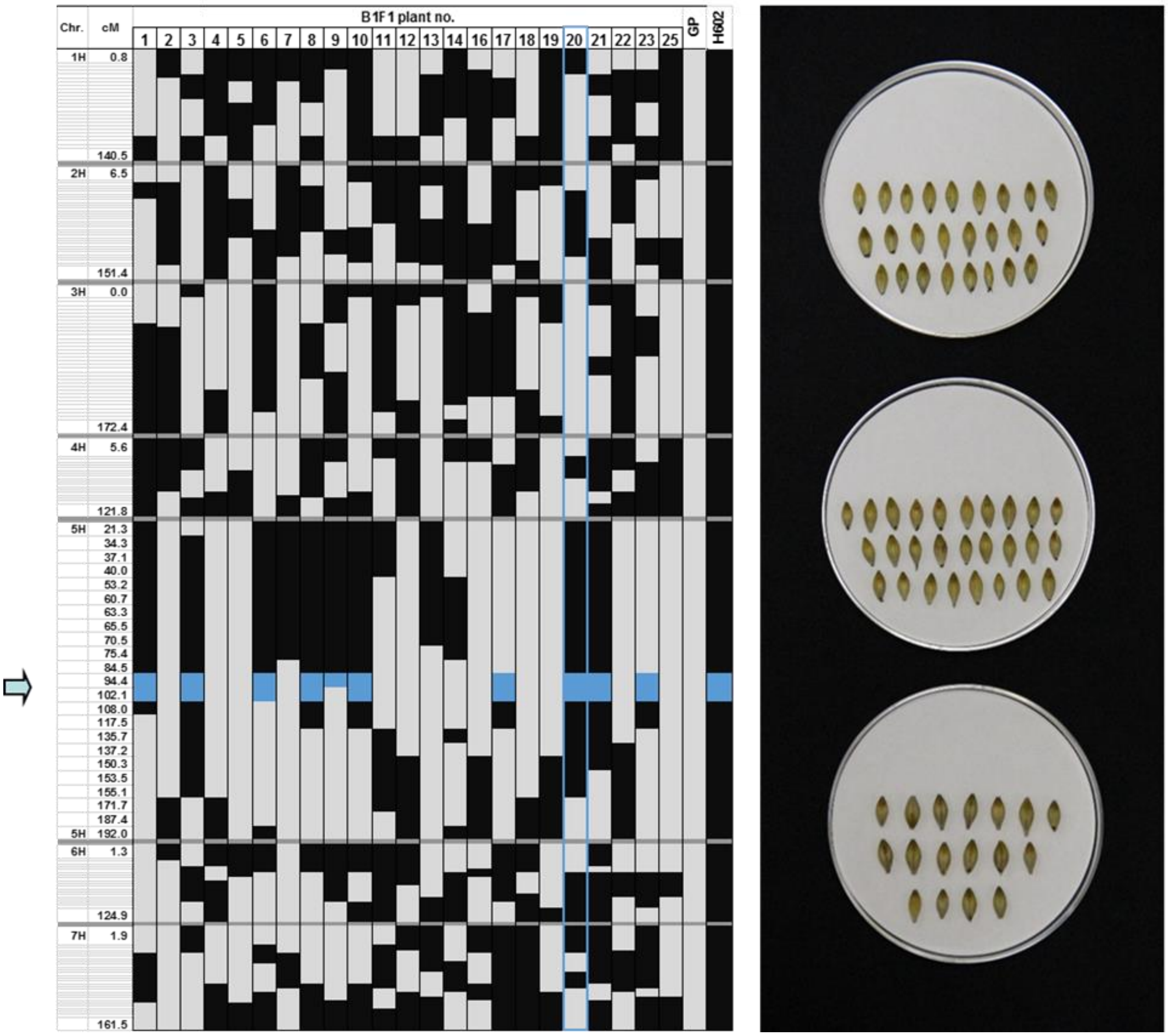

Supplementary Fig. 3| Genotyping and selection of B_1F_1 plants derived from the cross between GP (Golden Promise: transformation efficient with less dormant allele *Qsd1/Qsd1*) and H602 (transformation inefficient with dormant allele *qsd1/qsd1*). Left: Black box indicates heterozygous region of B_1F_1 plants on the background of GP (gray box). *Qsd1* region is indicated by arrow in blue region of each plant. The B_1F_1 plant No.20 (blue line) was self-pollinated to obtain a B_1F_2 *qsd1*/*qsd1* homozygous plant (B1H602GP20-7). Right: Seed samples of B1H602GP20-7 (each spike for each Petri dish) show no germination after 35 d of 25ºC dormancy reduction treatment.

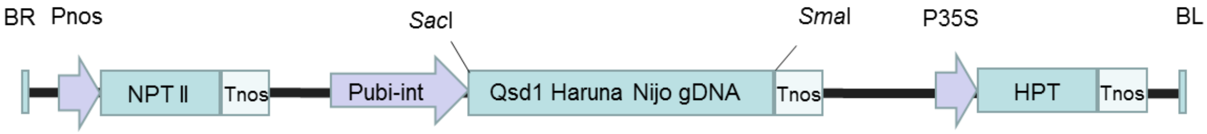

Supplementary Fig. 4| T-DNA region of pBUH3. Abbreviations are as follows: BR, right border; BL, left border; NPT II, neomycin; Phosphotransferase II; Pnos, nopaline synthase promoter; Tnos, 3' signal of nopaline synthase; P35S, 35S promoter; Pubi-int, maize ubiquitin promoter and the first intron; HPT, hygromycin phosphotransferase.

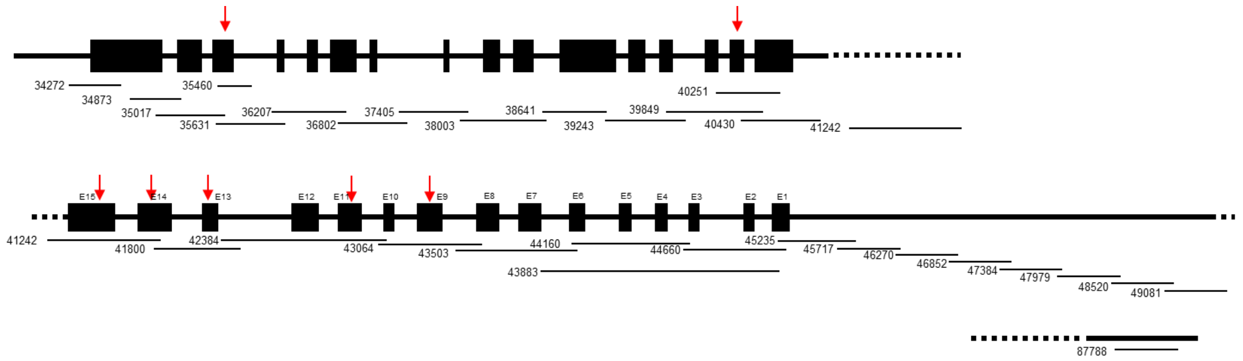

Supplementary Fig. 5| PCR amplification at the region of *LC054176* (upper) and *Qsd1* (lower). Arrows indicate SNPs in exon between parents (Haruna Nijo and H602).

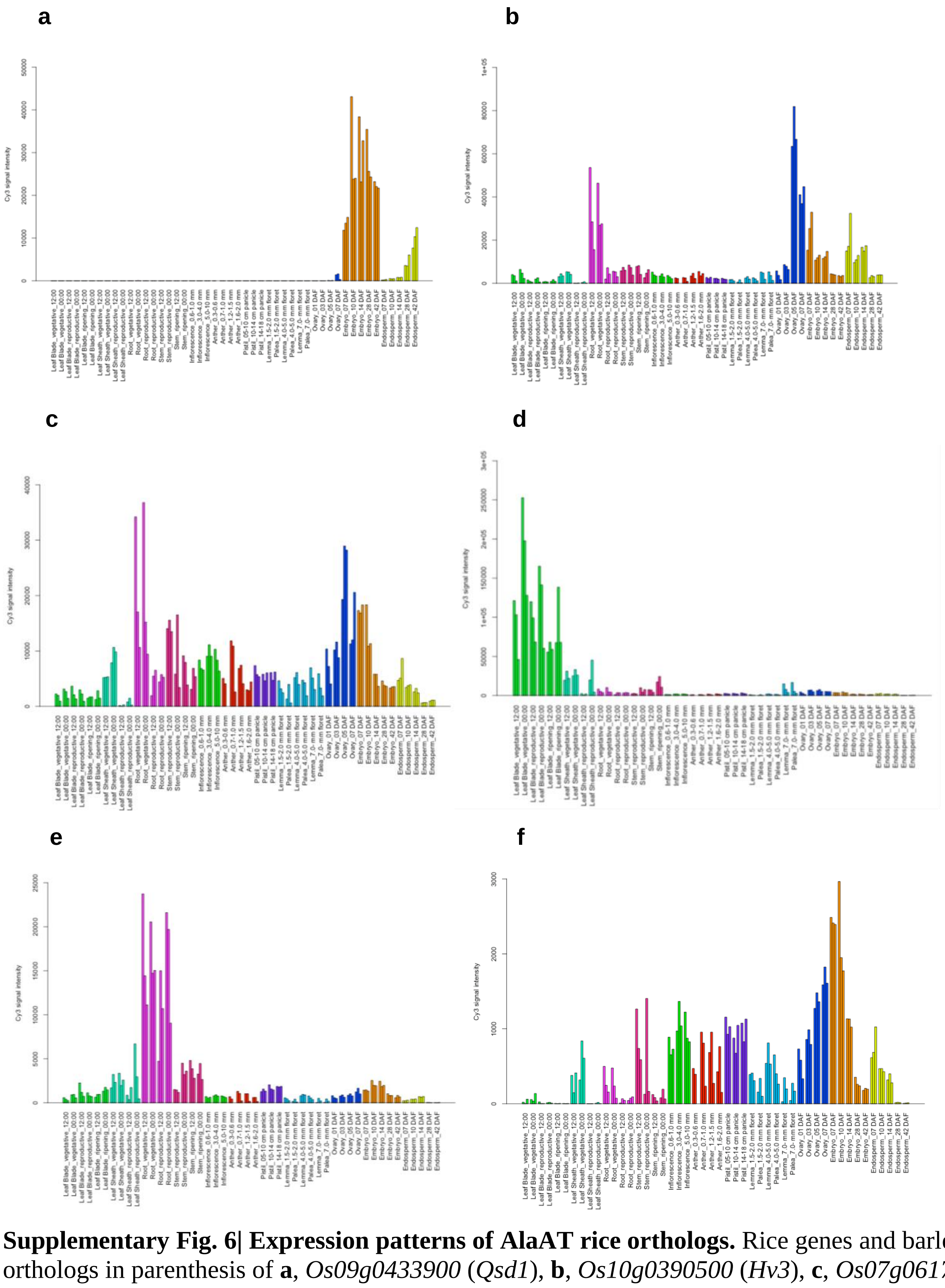

Supplementary Fig. 6| Expression patterns of AlaAT rice orthologs. Rice genes and barley orthologs in parenthesis of **a**, *Os09g0433900* (*Qsd1*), **b**, *Os10g0390500* (*Hv3*), **c**, *Os07g0617800* (*Hv2*), **d**, *Os07g0108300* (*Hv5*) and **e** and **f**, *Os10g0390600* (*Hv4*). *Os10g0390600* has two probes from full length cDNA *AK119373* (e) and *AK107029* (f) which might be derived from a different locus in RiceXPro[23].

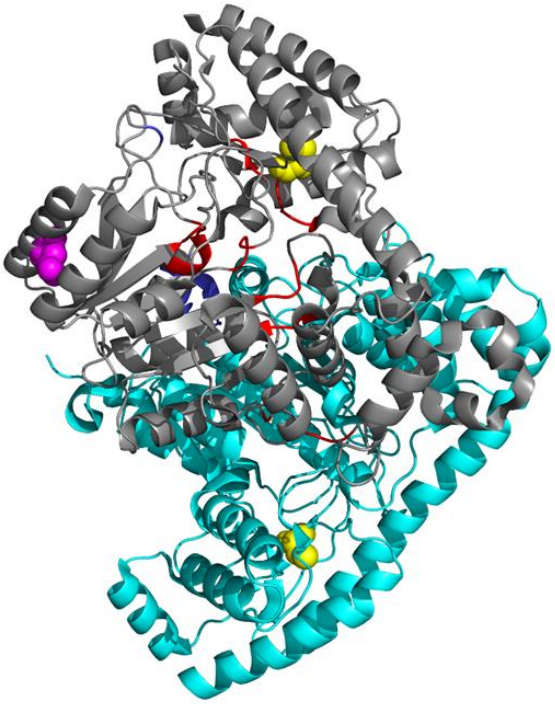

Supplementary Fig. 7| An *in silico* protein threaded model of QSD1 based on the crystal structure of AlaAT[24] (Hv3). The model contains two subunits, coloured grey and cyan. The predicted position of the L214F substitution is shown in magenta. The NH2-terminus is shown in blue, the pyrodixal 5’ phosphate binding pocket is in red, and cysteines are shown in yellow.

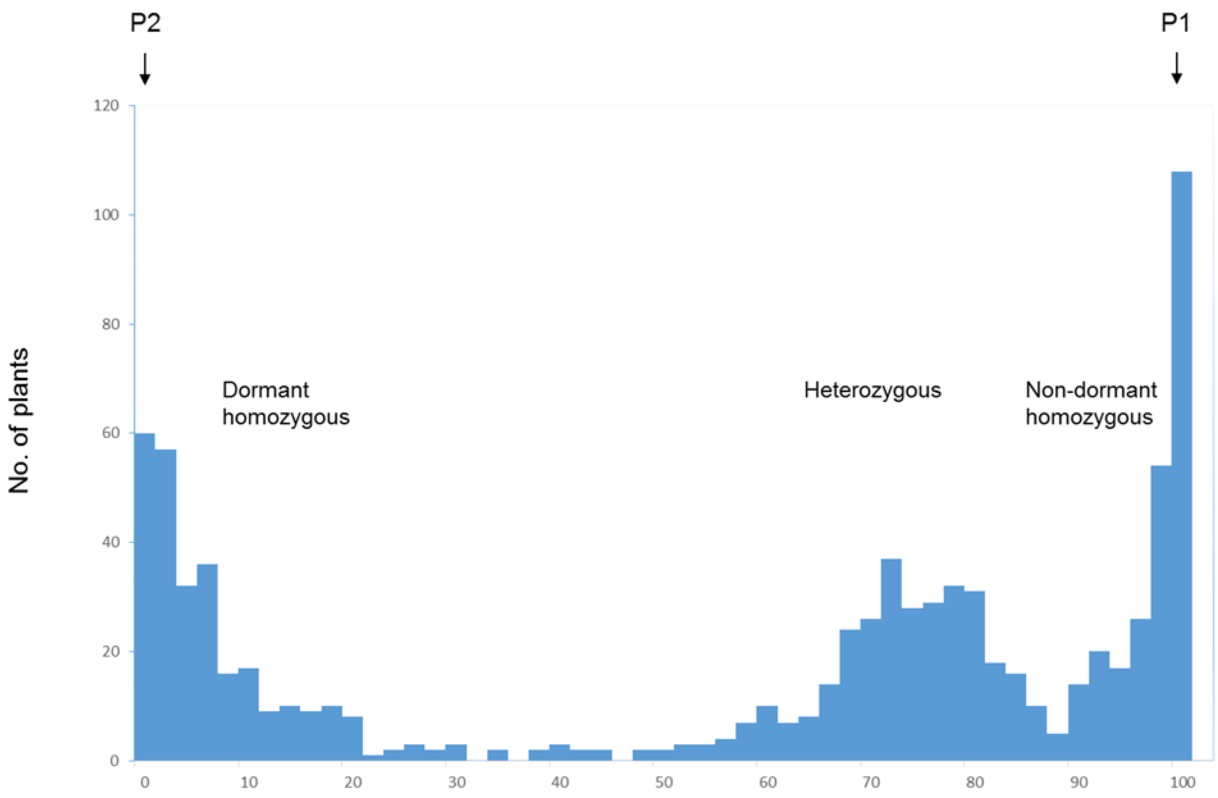

Supplementary Fig. 8| Frequency distribution of seed germination % in F_3 plants (F_4 seeds) from the cross Haruna Nijo (P1)/H602 (P2) after 28 d treatment of dormancy reduction. *Qsd1*/*qsd1* is genotyped by dormant homozygous, heterozygous and non-dormant homozygous.

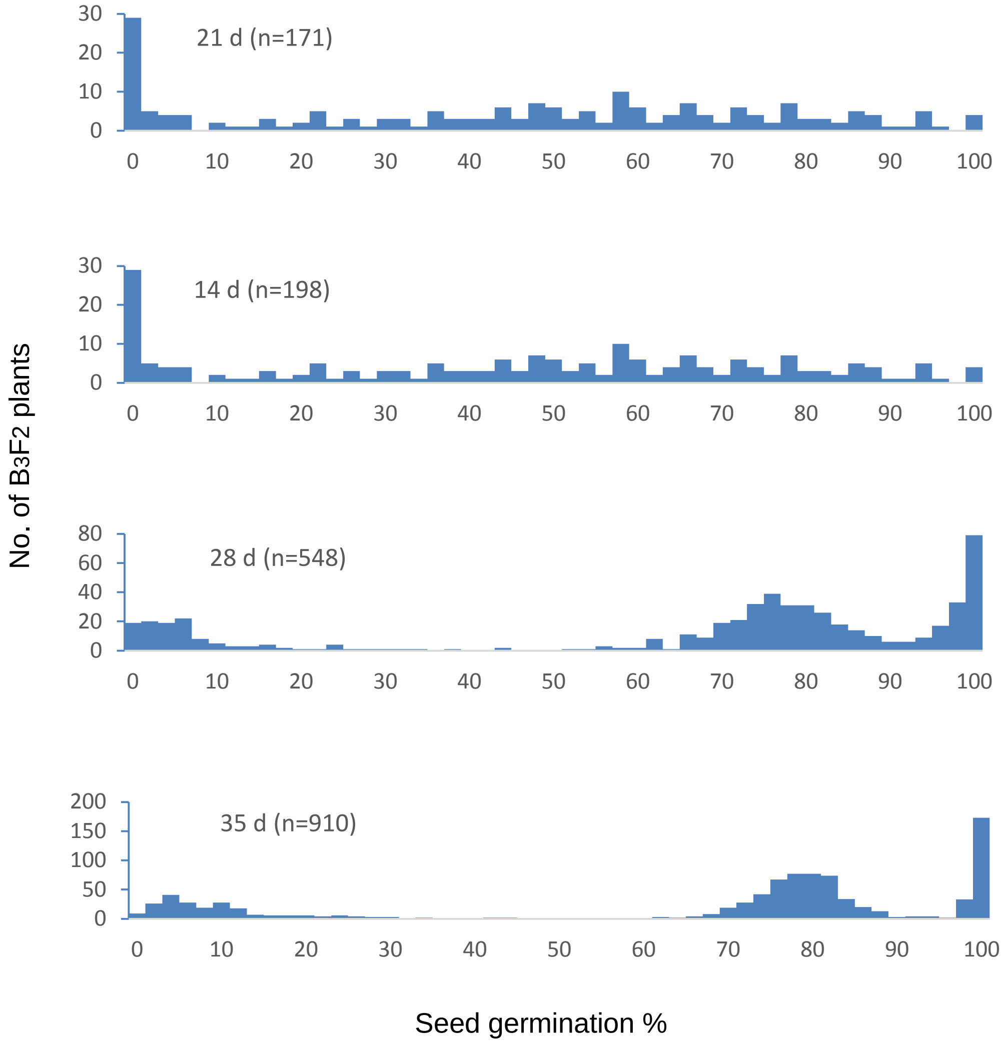

Supplementary Fig. 9| Frequency distributions of seed germination in F_2 plants (F_3 seeds) from the cross Haruna Nijo/H602 for time course treatments of dormancy reduction.

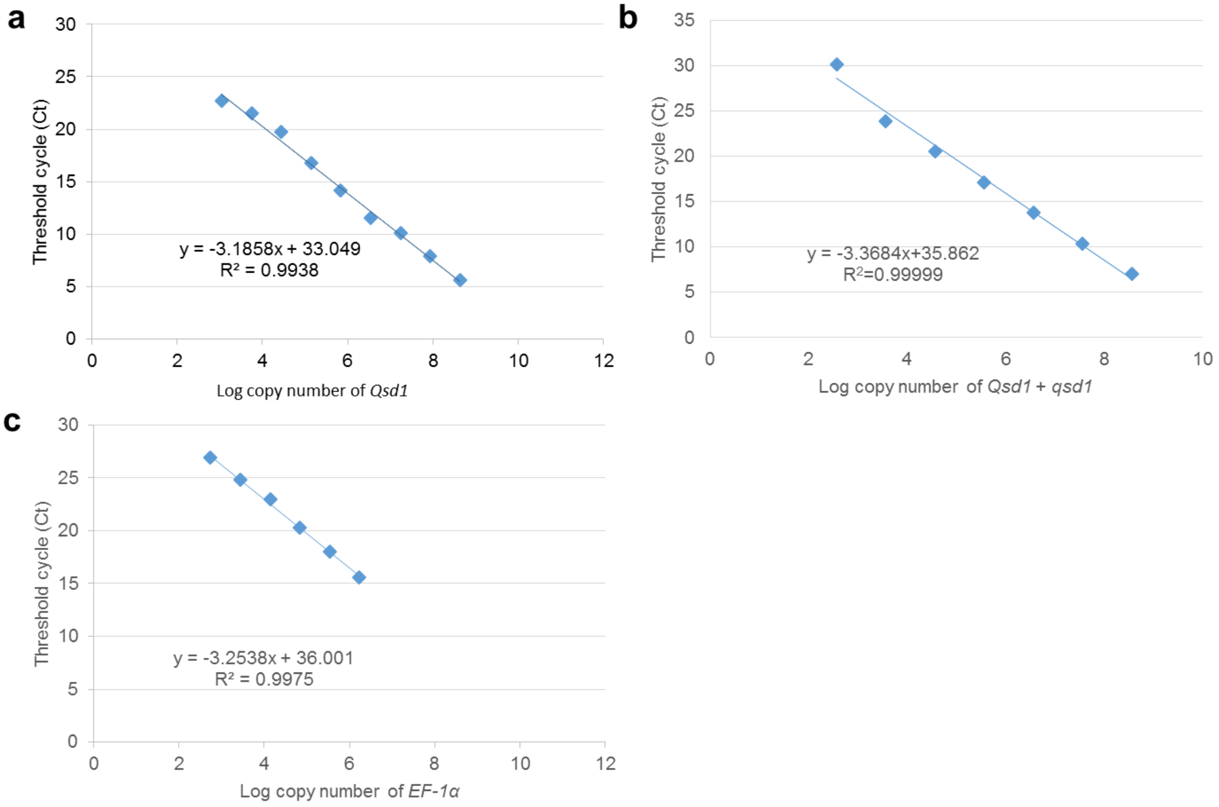

Supplementary Fig. 10| Log copy numbers for expression data. Each figure represents the expression for **a**, *qsd1* (H602), **b**, *Qsd1* and *qsd1* for complementation experiment and **c**, *EF1-α* as a control. For primer sequences, see Supplementary Table 8.

Supplementary Table 1| Primer information of markers for fine mapping of *Qsd1* region.

Primer name	sequence ID	sequence L	sequence R
EST1	k01353*	GCAACCGTAGTCCTTGTGGT	GTGTGGCCTTTCAGGTGATT
EST2	k04618*	ATCAGGCATATGACCCAAGG	AGGGAAATGGTGCACAAGAC
EST3	NIASHv3083L11_R**	CCGCTCCCCAAGAGTATTTC	GTTGCTAATCTCCCCGTTGT
EST4R	k00950*	ACTACAAAAGCCTTCCCCGT	TGCGAAACCATACCGTAACA
EST4F	FLOUbaf96g05_F	GTCCAATTCCCCACCACA	GCACGCAGCTCCTTGACA
EST5	NIASHv3013O02_F**	CAATCGCTCACTCTCCTCCT	TTCCGGTCTTGATGGAAAAC
EST6	CONTIG7699***	GTCGTCCTGAAGCAGATGGT	AACATGCACCCTACTTCAACG
EST7	NIASHv1145B20_R**	TACACTTCCGTGGCACAATC	GACGACGTGTACCTCGGCTA
EST8	NIASHv3118E04_R**	ATCACGGCAGGTGGTACAG	GTGTACCTCGGCTACGGCTA
EST9	NIASHv2005F14_F**	TTGCATTTCGTTCTCCAGTG	CATGAGCTACTGCACGTTCC
EST10	NIASHv3091J11_F**	CGCCCAACTAGCTAAAAACAG	CGACAGCGACTTCACGTCT
EST11	rbah40i16****	GAGATCAATGACGTGCTCCA	TGGGGAAGTTGTGTTGGAAT
EST12	k03227*	TGATTGTTACGTTGCCTGGA	CATGCCACATAATGCAAAGG

* Sato *et al.* [13]

** bex-db (http://barleyflc.dna.affrc.go.jp/bexdb/)

*** HarvEST (http://harvest.ucr.edu/) barley assembly #32

**** Barley DB (http://www.shigen.nig.ac.jp/barley/)

Supplementary Table 2| Nucleotide polymorphisms in the *Qsd1* region. Gene structures of *AK353915* and *LC054176* are tail-to-tail oriented to and separated only by 345 bp. Polymorphisms are indicated with E: exon and I: intron numbers and position refer to the Haruna Nijo BAC sequence (LC054174). NS show non synonymous amino acid change. Dormant and less-dormant parents used for QTL mapping[9] are compared.

			Non synonymous haplotype of *AK372829*				Gene structure of *LC054176*																									
E:exon, I:intron							E3	I3	I3	I3	I3	I5	E6	I6	I7	T7	I7	I8	I10	I10	E11	I11	I11	I11	E12	I12	I13	I13	I13	I14	I14	E15
Position on Haruna Nijo BAC clone LC054174							35654	35781	35812	36006	36032	36633	36795	36918	37510	37530	37762	37942	38684	38693	39301	39440	39451	39487	39558	39731	39954	40066	40086	40269	40272	40407
Qsd1 /*qsd1*	Acc. No.	Name																														
qsd1	H602	Wild barley	**T**	**G**	**C**	**C**	T	A	T	A	T	T	C	C	C	T	T	TCT	G	T	C	T	T	C	T	T	G	T	---	C	T	T
qsd1	I765	Khanaqin 1	**T**	**A**	**C**	**C**	T	A	T	A	T	T	C	C	C	T	T	TCT	G	T	C	T	T	C	T	T	G	T	---	C	T	T
qsd1	I767	Khanaqin 7	**T**	**A**	**C**	**C**	T	A	T	A	T	T	C	C	C	T	T	TCT	G	T	C	T	T	C	T	T	G	T	---	C	T	T
qsd1	T602	Turkey 6	**T**	**A**	**C**	**C**	T	A	T	A	T	T	C	C	C	T	T	TCT	G	T	C	T	T	C	T	T	G	T	---	C	T	T
qsd1	T615	Turkey 45	**T**	**A**	**C**	**C**	T	A	T	A	T	T	C	C	C	T	T	TCT	G	T	C	T	T	C	T	T	G	T	---	C	T	T
qsd1	I626	Katana 1	**C**	**A**	**T**	**C**	C	A	T	G	C	G	T	C	T	C	C	---	C	C	T	C	T	A	C	C	G	C	CTT	C	T	C
qsd1	I622	H. E. S. 4	**C**	**A**	**T**	**C**	C	-	T	G	T	T	C	T	C	C	C	---	C	C	C	C	T	A	T	C	A	T	CTT	C	T	C
qsd1	C627	Mokussekko 3	**C**	**A**	**T**	**C**	C	A	T	G	T	T	C	T	C	C	C	---	C	C	C	C	T	A	T	C	G	T	CTT	T	T	C
qsd1		Steptoe	**C**	**A**	**T**	**C**	C	A	C	G	T	T	C	C	C	C	C	---	C	C	C	C	C	A	T	C	G	C	CTT	C	T	C
Qsd1		Morex	**C**	**A**	**T**	**G**	C	-	T	G	T	T	C	T	C	C	C	---	C	C	C	C	T	A	T	C	A	T	CTT	C	C	C
Qsd1	J215	Ko A	**C**	**A**	**T**	**G**	C	-	T	G	T	T	C	T	C	C	C	---	C	C	C	C	T	A	T	C	A	T	CTT	C	C	C
Qsd1	U062	Russia 6	**C**	**A**	**T**	**G**	C	-	T	G	T	T	C	T	C	C	C	---	C	C	C	C	T	A	T	C	A	T	CTT	C	C	C
Qsd1	C649	Harbin 2-row	**C**	**A**	**T**	**G**	C	-	T	G	T	T	C	T	C	C	C	---	C	C	C	C	T	A	T	C	A	T	CTT	C	C	C
Qsd1	J247	Haruna Nijo	**C**	**A**	**T**	**G**	C	-	T	G	T	T	C	T	C	C	C	---	C	C	C	C	T	A	T	C	A	T	CTT	C	C	C

			Intergenic			Gene structure of *AK372829*																										
E:exon, I:intron						E15	E15	E15	I14	I14	**E14**	I13	**E13**	I12	I12	**E11**	**E9**	I8	E8	I6	I5	I5	I3	I2	I2	I2	I2	I2	I2	I2	I2	I1
Position on Haruna Nijo BAC clone LC054174			40947	41105	41169	41331	41490	41535	41641	41667	**41771**	41916	**42095**	42152	42521	**42950**	**43368**	43574	43654	44044	44295	44362	44675	44791	44846	44853	44870	44879	44909	44912	45025	45109
Qsd1 /*qsd1*	Acc. No.	Name																														
qsd1	H602	Wild barley	G	T	G	--	C	G	---	A	**T**	CA	**G**	-	C	**C**	**C**	T	A	C	C	CT	A	C	A	G	T	A	TA	A	T	A
qsd1	I765	Khanaqin 1	G	T	G	--	C	G	---	A	**T**	CA	**A**	-	C	**C**	**C**	T	A	C	C	CT	A	C	A	G	T	A	TA	A	T	A
qsd1	I767	Khanaqin 7	G	T	G	--	C	G	---	A	**T**	CA	**A**	-	C	**C**	**C**	T	A	C	C	CT	A	C	A	G	T	A	TA	A	T	A
qsd1	T602	Turkey 6	G	T	G	--	C	G	---	A	**T**	CA	**A**	-	C	**C**	**C**	T	A	C	C	CT	A	C	A	G	T	A	TA	A	T	A
qsd1	T615	Turkey 45	G	T	G	--	C	G	---	A	**T**	CA	**A**	-	C	**C**	**C**	T	A	C	C	CT	A	C	A	G	T	A	TA	A	T	A
qsd1	I626	Katana 1	G	A	G	GA	T	T	AAC	A	**C**	CA	**A**	C	A	**T**	**C**	C	A	A	T	TT	G	T	T	T	C	G	--	G	T	G
qsd1	I622	H. E. S. 4	C	T	G	GA	C	T	AAC	A	**C**	AT	**A**	C	A	**T**	**C**	C	A	C	C	CT	G	T	T	T	C	G	--	G	T	A
qsd1	C627	Mokussekko 3	C	T	G	GA	T	T	AAC	G	**C**	CA	**A**	C	A	**T**	**C**	C	A	C	T	CT	G	T	T	T	C	G	--	G	T	A
qsd1		Steptoe	G	A	A	GA	C	T	AAC	A	**C**	CA	**A**	C	A	**T**	**C**	C	G	C	C	CA	G	T	T	T	C	G	--	G	C	A
Qsd1		Morex	C	T	G	GA	C	T	AAC	A	**C**	AT	**A**	C	A	**T**	**G**	C	A	C	C	CT	G	T	T	T	C	G	--	G	T	A
Qsd1	J215	Ko A	C	T	G	GA	C	T	AAC	A	**C**	AT	**A**	C	A	**T**	**G**	C	A	C	C	CT	G	T	T	T	C	G	--	G	T	A
Qsd1	U062	Russia 6	C	T	G	GA	C	T	AAC	A	**C**	AT	**A**	C	A	**T**	**G**	C	A	C	C	CT	G	T	T	T	C	G	--	G	T	A
Qsd1	C649	Harbin 2-row	C	T	G	GA	C	T	AAC	A	**C**	AT	**A**	C	A	**T**	**G**	C	A	C	C	CT	G	T	T	T	C	G	--	G	T	A
Qsd1	J247	Haruna Nijo	C	T	G	GA	C	T	AAC	A	**C**	AT	**A**	C	A	**T**	**G**	C	A	C	C	CT	G	T	T	T	C	G	--	G	T	A
											**NS**		**NS**			**NS**	**NS**															

Supplementary Table 3| Degree of less-dormancy (% of germination) of mapping parents used for QTL detection and additive effects of *Qsd1* in each population[9].

Name	Accession No.	Allele	Non-synonymous SNP				Germination (%) after 25ºC treatment		Additive effect of *Qsd1* allele (germination %)		Cross and *Qsd1* donor
			E14	E13	E11	E9	5w	10w	5w	10w	
Wild barley	H602	*qsd1*	T	G	C	C	0	0	15.8	26.5	*J247* /H602
Khanaqin 1	I765	*qsd1*	T	A	C	C	0	1	48.1	23.6	*C649* /I765
Khanaqin 7	I767	*qsd1*	T	A	C	C	0	0	46.2	42.5	*C649* /I767
Turkey 6	T602	*qsd1*	T	A	C	C	0	0	32.3	44.9	*C649* /T602
Turkey 45	T615	*qsd1*	T	A	C	C	0	0	29.8	45.7	*C649* /T615
Katana 1	I626	*qsd1*	C	A	T	C	12	24	13.1	10.6	*C649* /I626
H. E. S. 4	I622	*qsd1*	C	A	T	C	27	-	11.4	-	*U602* /I622
Mokussekko 3	C627	*qsd1*	C	A	T	C	4	-	27.5	-	*J215* /C627
Ko A	J215	*Qsd1*	C	A	T	G	91	-	0	-	*J215*
Russia 6	U062	*Qsd1*	C	A	T	G	93	-	0	-	*U062*
Harbin 2-row	C649	*Qsd1*	C	A	T	G	100	100	0	0	*C649*
Haruna Nijo	J247	*Qsd1*	C	A	T	G	100	100	0	0	*J247*

Supplementary Table 4| Association between haplotypes of non-synonymous SNPs in *Qsd1/qsd1* and average seed germination among 353 cultivated and 14 wild barley accessions.

Non-synonymous SNP				Wild		Cultivated		Reference
E14	E13	E11	E9	%*	n	%*	n	
T	G	C	C	0.0	1			H602
T	A	C	C	1.5	4	5.5	4	
C	A	C	C			40.6	7	
C	A	T	C	0.0	6	56.2	235	
T	A	T	C	0.0	3	62.0	61	
C	A	C	G			80.1	17	
C	A	T	G			83.0	29	Haruna Nijo

*Average germination % after 35 d of 25ºC dormancy reduction treatment

Supplementary Table 5| Primer information for polymorphism detection on *Qsd1* region.
Position is based on the numbers from 3' end of Haruna Nijo BAC clone (LC054174).

Primer name	Position on BAC clone LC054174			Product size	Sequence L	Sequence R
34272	34272	-	34778	507	CCCGAAATCCCGTCTAGTCT	AAGATAGAAGCCGACCAAACC
34873	34873	-	35372	500	GTCCAATTCCCCACCACA	GCACGCAGCTCCTTGACA
35017	35017	-	35698	698	CTTCTCCCCCACGCTTTACT	ACGAGCGAACCAATCTCCT
35460	35460	-	35974	515	CGTCCTATCCGTTTCCTGAG	TGGACAAGTTTCTTCAGATTCA
35631	35631	-	36300	670	GTCGTGCAACCCAACTTCTT	TGCAAAGAATAATGTGTCTACCTTT
36207	36207	-	36836	656	TGGCTTGGTTATAACACAGGTCTAT	ATATCTTTATGGCTGGGGACA
36802	36802	-	37446	671	GAAATGGCTGAGGAAGAAGG	AACTGAGTGAACTCAATCTGTGTATCT
37405	37405	-	38071	691	CATGATGTGCGGCACTAAAC	TTGGGAGCAGAAAAGAAACTG
38003	38003	-	38682	682	GAGCCTTTGTTGGTAGAGTTGC	CATGCATAAACAATGCGAATAGA
38641	38641	-	39330	690	TTGCCATCGCTTGGTTACT	GAGATGGCCCTGAAGTTGAT
39243	39243	-	39935	693	CCAAACTTGCCATCTGCTC	CCCTTAGTTCCTCAGCATTGA
39849	39849	-	40530	690	ATGACCGTACGTATCCACTATTCA	CATCTGTCTTCGCGTAATCCT
40251	40251	-	40763	513	TTTGCTTTGTCACAGGTTCG	AAAAGCCTTCCCCGTCTCTA
40430	40430	-	41086	657	TGGGCACTTTCAAGGTAAAAA	CAAAACTGAGGTCAATGTACGC
41242	41242	-	41866	625	GGGAGGCACTCACTCAGTACA	AGGGAATGACCTGCAACAAA
41800	41800	-	42337	538	GCTGCTGGGAGATGGATG	CCCACTGTTTCGCATACTTG
42384	42384	-	43138	755	TGCAGCAAAAGTCAACAAGG	TCGGAAAGAAGGCTTGGTTA
43064	43064	-	43656	593	CGCCCGCGTCACAAAACTAC	GGTGGGACTATGGTATGGTGG
43503	43503	-	44194	692	ACGCACTCCCACTTTACACC	ATTGCAGTCCATGTGCCATA
43883	43883	-	45247	1364	CGCCACCTTAGCTAGCTCTC	ATGTCTTCCGGTCTTGATGG
44160	44160	-	44722	563	CTTCCGCGCTCTATTTATGG	CCAGAGGTGCGTCTTTCTTT
44660	44660	-	45294	635	GCTCTGGAAGTCTGGGTTGA	CAGCACAATCGCTCACTCTC
45235	45235	-	45835	601	GGGAGGAAGGAGGAGAGCTA	AAACATGCGTGGAAGGTCAT
45717	45717	-	46366	649	CGATATAAATTATGCAAACATGACG	CGACAATCGTGAAGGTTTGAG
46270	46270	-	46923	653	CTCTCCTCCTTCGACACGAT	ATCCGCACCACACTACATCA
46852	46852	-	47504	652	GACGGTGAGCGAAGAAGG	CGAGGGGAGTAGTATACATCTAAAGC
47384	47384	-	48042	658	TCGTGCGGATGATAACCATA	CCAAGTCTTGCTGGGTTGTT
47949	47949	-	48639	690	CAGCGGAGGGTGAAACATAC	TACCGGACGACTGGACCTAC
48520	48520	-	49204	684	TTGGTTTCCTCCACCTCTTG	GTCGAGGAGGCCGAACTT
49081	49081	-	49742	661	CTACGCCCTCGGCCTCTT	GGCCGTAAGACCCCTGACC
87788	87788	-	88452	664	CAGATTGTTCGGGGGTACTG	GAGAAACACACCGGTTGAAAA

Supplementary Table 6| Non-redundant haplotypes of 27 wild and 210 cultivated barleys on *Qsd1*/*qsd1*.

I:intron E:exon	E15	E15	E15	E15	E15	E15	E15	E15	E15	I14	I14	E14	E14	E14	I13	I13	I13	I13	I13	I13	E13	I12	I12	I12	I12	I12	I12	I12	I12	I12	I12	I12	I12	I12
Nucleotide position on BAC HN_550I12 contig3	41331-41332	41384	41392	41395	41404	41490	41535	41542	41563	41640-41642	41667	41735	41750	41771	41916-41917	41969	41977	41993	42006	42007	42095	42152	42161	42180	42182	42271	42491	42512	42521	42563	42587	42601	42616	42640
W_H602	--	C	G	G	G	C	G	C	G	---	A	C	C	T	CA	T	C	T	---	A	G	-	C	A	G	A	C	C	C	T	T	G	A	C
W387	.	.	.	.	.	.	.	.	.	.	.	.	.	.	.	.	.	.	.	.	.	.	.	.	.	.	.	.	.	.	.	.	.	.
W395	.	.	.	.	.	.	.	.	.	.	.	.	.	.	.	.	.	.	.	.	.	.	.	.	.	.	.	.	.	.	.	.	.	.
WC394	.	.	.	.	.	.	.	.	.	.	.	.	.	.	.	.	.	.	.	.	A	.	.	.	.	.	.	.	.	.	.	.	.	.
W393	.	.	.	.	.	.	.	.	.	.	.	.	.	.	.	.	.	.	.	.	A	.	.	.	.	.	.	.	.	.	.	.	.	.
C064	GA	.	.	.	.	.	.	.	.	AAC	.	.	.	.	.	.	.	.	.	.	A	C	.	.	.	G	.	T	.	C	.	.	.	.
C155	GA	.	.	.	.	.	.	.	.	AAC	.	.	.	.	.	.	.	.	.	.	A	C	.	.	.	G	.	T	.	C	.	.	.	.
C211	GA	.	.	.	.	.	.	.	.	AAC	.	.	.	.	.	.	.	.	.	.	A	C	.	.	.	G	.	T	.	C	.	.	.	.
C378	GA	.	.	.	.	.	T	.	.	AAC	.	.	.	C	.	.	.	.	.	.	A	C	.	.	.	.	.	.	A	.	.	.	.	.
C351	GA	.	.	.	.	.	T	.	.	AAC	.	.	.	C	.	.	.	.	.	.	A	C	.	.	.	.	.	.	A	.	.	.	.	.
W388	GA	.	.	.	.	.	T	.	.	AAC	.	.	.	C	.	.	.	.	.	.	A	C	.	.	.	.	.	.	A	.	.	.	.	.
C075	GA	.	.	.	.	.	T	.	.	AAC	.	.	.	C	.	.	.	.	.	.	A	C	.	.	.	.	.	.	A	.	.	.	.	.
W382	GA	.	.	.	.	.	T	.	.	AAC	.	.	.	C	.	.	.	.	.	.	A	C	.	.	.	.	.	.	A	.	.	.	.	.
C153	GA	.	.	.	.	.	T	.	.	AAC	.	.	.	C	.	.	.	.	.	.	A	C	.	.	.	.	.	.	A	.	.	.	.	.
C011	GA	.	.	.	.	.	T	.	.	AAC	.	.	.	C	.	.	.	.	.	.	A	C	.	.	.	.	.	.	A	.	.	.	.	.
C025	GA	.	.	.	.	.	T	.	.	AAC	.	.	.	C	.	.	.	.	.	.	A	C	.	.	.	.	.	.	A	.	.	.	.	.
C126	GA	.	.	.	.	.	T	.	.	AAC	.	.	.	C	.	.	.	.	.	.	A	C	.	.	.	.	.	.	A	.	.	.	.	.
C021	GA	.	.	.	.	.	T	.	.	AAC	.	.	.	C	.	.	.	.	.	.	A	C	.	.	.	.	.	.	A	.	.	.	.	.
W391	GA	.	.	.	.	.	T	.	.	AAC	.	.	.	C	.	.	.	.	.	.	A	C	.	.	.	.	.	.	A	.	.	.	.	.
C030	GA	.	.	.	.	.	T	.	.	AAC	.	.	.	C	.	.	A	.	.	.	A	C	.	.	.	.	.	.	A	.	.	.	.	.
C361	GA	.	.	.	.	.	T	.	.	AAC	.	.	.	C	AT	.	.	.	.	.	A	C	.	.	.	.	.	.	A	.	.	.	.	.
C353	GA	.	.	.	.	.	T	.	.	AAC	.	.	.	C	AT	.	.	.	.	.	A	C	.	.	.	.	.	.	A	.	.	.	.	.
C154	GA	.	.	.	.	.	T	.	.	AAC	.	.	.	C	AT	.	.	.	.	.	A	C	.	.	.	.	.	.	A	.	.	.	.	.
C302	GA	.	.	.	.	.	T	.	.	AAC	.	.	.	C	AT	.	.	.	.	.	A	C	.	.	.	.	.	.	A	.	.	.	.	.
WC398	GA	.	.	.	.	.	T	.	.	AAC	.	.	.	C	AT	.	.	.	.	.	A	C	.	.	.	.	.	.	A	.	.	.	.	.
C317	GA	.	.	.	.	.	T	.	.	AAC	.	.	.	C	AT	.	.	.	.	.	A	C	.	.	.	.	.	.	A	.	.	.	.	.
C350	GA	.	.	.	.	.	T	.	.	AAC	.	.	.	C	AT	.	.	.	.	.	A	C	.	.	.	.	.	.	A	.	.	.	.	.
C220	GA	.	.	.	.	.	T	.	.	AAC	.	.	.	C	AT	.	.	.	.	.	A	C	.	.	.	.	.	.	A	.	.	.	.	.
C_HN	GA	.	.	.	.	.	T	.	.	AAC	.	.	.	C	AT	.	.	.	.	.	A	C	.	.	.	.	.	.	A	.	.	.	.	.
C266	GA	.	.	.	.	.	T	.	.	AAC	.	.	.	C	AT	.	.	.	.	.	A	C	T	.	.	.	.	.	A	.	.	.	.	.
C043	GA	.	.	.	.	.	T	.	.	AAC	.	.	.	C	AT	.	.	.	.	.	A	C	T	.	.	.	.	.	A	.	.	.	.	.
C308	GA	.	.	.	.	.	T	.	.	AAC	.	.	T	C	AT	.	.	.	.	.	A	C	.	.	.	.	.	.	A	.	.	.	.	.
C325	GA	.	.	.	.	.	T	.	.	AAC	.	.	T	C	AT	.	.	.	.	.	A	C	.	.	.	.	.	.	A	.	.	.	.	.
W386	GA	.	.	.	.	T	.	.	.	AAC	.	.	.	.	.	.	.	.	.	.	A	C	.	.	.	.	.	.	.	.	.	.	.	.
WC051	GA	.	.	.	.	T	T	.	.	AAC	G	.	.	C	.	.	.	.	.	.	A	C	.	.	.	.	.	.	A	.	.	.	.	.
C172	GA	.	.	.	.	T	T	.	.	AAC	G	.	.	C	.	.	.	.	.	.	A	C	.	.	.	.	.	.	A	.	.	.	.	.
C063	GA	.	.	.	.	T	T	.	.	AAC	G	.	.	C	.	.	.	.	.	.	A	C	.	.	.	.	.	.	A	.	.	.	.	.
C125	GA	.	.	.	.	T	T	.	.	AAC	G	T	.	C	.	.	.	.	.	.	A	C	.	.	.	.	.	.	A	.	.	.	.	.
C121	GA	T	A	.	.	.	.	.	.	AAC	.	.	.	.	.	A	.	.	.	G	A	C	.	.	.	.	.	.	.	.	.	.	.	.
W0303	GA	.	.	.	.	.	T	.	.	AAC	.	.	.	C	AT	.	.	.	.	.	A	C	.	.	.	.	.	.	A	.	.	.	.	.
W0982	GA	.	.	.	A	.	.	.	.	AAC	.	.	.	.	.	.	.	.	.	.	A	C	.	.	.	.	.	.	.	.	.	.	.	.
W1215	GA	.	.	.	.	.	T	.	.	AAC	.	.	.	C	AT	.	.	.	.	.	A	C	.	.	.	.	.	.	A	.	.	A	.	.
W1238	.	.	.	.	.	.	.	.	.	.	.	.	.	.	.	.	.	.	.	.	A	-	.	.	.	.	.	.	.	.	.	.	.	.
W1277	GA	.	.	.	.	.	.	.	.	AAC	.	.	.	.	.	.	.	.	.	.	A	C	.	.	.	G	.	.	.	C	.	.	.	T
W1306	.	.	.	.	.	.	.	.	.	AAC	.	.	.	.	.	.	.	.	.	.	A	-	.	.	.	.	.	.	.	.	.	.	.	.
W1393	GA	.	.	.	.	.	T	.	.	AAC	.	.	.	C	.	.	.	C	.	.	A	C	.	.	.	.	.	.	A	.	C	.	.	.
W1440	GA	.	.	.	.	.	T	.	.	AAC	.	.	.	C	.	.	.	.	.	.	A	C	.	.	.	.	.	.	A	.	.	.	.	.
W1454	GA	.	.	.	.	.	.	.	.	AAC	.	.	.	.	.	.	.	.	.	.	A	C	.	.	.	G	.	.	.	C	.	.	.	T
W1466	GA	.	.	.	.	.	T	.	.	AAC	.	.	.	C	.	.	.	.	.	.	A	C	.	.	.	.	.	.	A	.	.	.	.	.
W1488	GA	.	.	.	A	.	.	A	T	AAC	.	.	.	.	.	.	.	.	.	.	A	C	.	.	A	.	.	.	.	.	.	.	.	.
W1573	.	.	.	A	.	.	.	.	.	AAC	.	.	.	.	.	.	.	.	.	.	A	C	.	.	.	.	.	.	.	.	.	.	.	.
W1585	GA	.	.	.	.	.	T	.	.	AAC	.	.	.	C	AT	.	.	.	.	.	A	C	.	.	.	.	.	.	A	.	.	.	.	.
W1632	GA	.	.	.	.	.	.	.	.	AAC	.	.	.	.	.	.	.	.	G	.	A	C	.	G	.	.	.	.	.	.	.	.	G	.
W1725	GA	.	.	.	.	.	T	.	.	AAC	.	.	.	C	.	.	.	.	.	.	A	C	.	.	.	.	.	.	A	.	.	.	.	.

I:intron E:exon	I11	I11	E11	E11	I11	E10	I9	I9	I9	E9	E9	I8	I8	I8	I8	I8	E8	E8	E8	I7	E7	I6	I6	I6
Nucleotide position on BAC HN_550I12 contig3	42872	42888	42941	42950	43067	43135	43210	43232	43235	43299	43368	43442	43558	43562	43574	43575	43654	43672	43681	43816	43942	43971	44019	44044
W_H602	T	T	T	C	C	C	G	A	T	T	C	G	T	A	T	T	A	C	T	G	G	A	T	C
W387	.	.	.	.	.	.	.	.	.	.	.	.	.	.	.	.	.	.	.	.	.	.	.	.
W395	.	.	.	.	.	.	.	.	.	.	.	.	.	.	.	.	.	.	.	.	.	.	.	.
WC394	.	.	.	.	.	.	.	.	.	.	.	.	.	.	.	.	.	.	.	.	.	.	.	.
W393	.	.	.	.	.	.	.	.	.	.	G	.	.	.	C	.	.	.	.	.	.	.	.	.
C064	.	.	.	T	.	.	.	C	-	C	.	C	.	.	C	A	.	.	.	.	.	.	.	.
C155	.	.	.	T	.	G	.	C	-	C	.	.	.	.	C	.	.	.	.	.	.	.	.	A
C211	.	.	.	T	.	G	.	C	-	C	.	C	.	.	C	A	.	.	.	.	.	.	.	A
C378	.	.	.	.	.	.	.	.	.	.	.	.	.	.	C	.	.	.	.	.	.	.	.	.
C351	.	.	.	.	.	.	.	.	.	.	.	.	.	.	C	.	G	.	.	.	.	.	.	.
W388	.	.	.	T	.	.	.	.	.	.	.	.	.	.	.	.	.	.	.	.	.	.	.	.
C075	.	.	.	T	.	.	.	.	.	.	.	.	.	.	C	.	.	.	.	.	.	.	.	.
W382	.	.	.	T	.	.	.	.	.	.	.	.	.	.	C	.	.	.	.	.	.	.	.	.
C153	.	.	.	T	.	.	.	.	.	.	.	.	.	.	C	.	.	.	.	.	.	.	.	.
C011	.	.	.	T	.	.	.	.	.	.	.	.	.	.	C	.	.	.	.	.	.	.	-	.
C025	.	.	.	T	.	.	.	.	.	.	.	.	.	.	C	.	.	.	.	.	.	.	-	.
C126	.	.	.	T	.	.	.	.	.	.	.	.	.	.	C	.	G	.	.	.	.	.	.	.
C021	.	.	.	T	.	.	.	.	.	.	.	.	.	.	C	.	G	.	.	.	.	.	.	.
W391	.	.	.	T	.	.	.	.	.	.	.	.	C	.	.	.	.	.	.	.	.	.	.	.
C030	.	.	.	T	.	.	.	.	.	.	.	.	.	.	C	.	.	.	.	.	.	.	-	.
C361	.	.	.	.	.	.	.	.	.	.	.	.	.	.	C	.	.	.	.	.	.	.	.	.
C353	.	.	.	.	.	.	.	.	.	.	G	.	.	.	C	.	.	.	.	.	.	.	.	.
C154	.	.	.	T	.	.	.	.	.	.	.	.	.	.	C	.	.	.	.	.	.	.	.	.
C302	.	.	.	T	.	.	.	.	.	.	.	.	.	.	C	.	.	.	.	.	.	.	.	.
WC398	.	.	.	T	.	.	.	.	.	.	.	.	.	.	C	.	.	.	.	.	.	.	.	.
C317	.	.	.	T	.	.	.	.	.	.	.	.	.	.	C	.	.	.	.	.	.	.	-	.
C350	.	.	.	T	.	.	.	.	.	.	.	.	.	.	C	.	.	.	.	.	.	.	.	A
C220	.	.	.	T	.	.	.	.	.	.	G	.	.	.	C	.	.	.	.	.	.	.	.	.
C_HN	.	.	.	T	.	.	.	.	.	.	G	.	.	.	C	.	.	.	.	.	.	.	.	.
C266	.	.	.	T	.	.	.	.	.	.	.	.	.	.	C	.	.	.	.	.	.	.	.	.
C043	.	.	.	T	.	.	.	.	.	.	.	.	.	.	C	.	.	.	.	.	.	.	.	.
C308	.	.	.	T	.	.	.	.	.	.	.	.	.	.	C	.	.	.	.	.	.	.	.	.
C325	.	.	.	T	.	.	.	.	.	.	.	.	.	.	C	.	.	.	.	.	.	.	.	.
W386	.	.	.	T	.	.	.	.	.	.	.	.	.	.	C	.	G	.	.	.	.	.	.	.
WC051	.	.	.	T	.	.	.	.	.	.	.	.	.	.	C	.	.	.	.	.	.	.	.	.
C172	.	.	.	T	.	.	.	.	.	.	.	.	.	.	C	.	.	.	.	.	.	.	.	.
C063	.	.	.	T	.	G	.	.	.	.	.	.	.	.	C	.	.	.	.	.	.	.	.	.
C125	.	.	.	T	.	.	.	.	.	.	.	.	.	.	C	.	.	.	.	.	.	.	.	.
C121	.	.	.	T	T	G	C	.	-	.	.	C	.	.	C	A	.	.	.	.	.	.	.	.
W0303	.	.	.	T	.	.	.	.	.	.	.	.	.	.	C	.	.	.	.	.	.	.	.	.
W0982	.	.	.	T	.	G	.	.	-	.	.	C	.	.	C	A	.	.	C	.	.	.	.	.
W1215	.	.	.	T	.	.	.	.	.	.	.	.	.	.	C	.	.	.	.	.	.	.	.	.
W1238	.	.	.	.	.	.	.	.	.	.	.	.	.	.	.	.	.	.	.	.	.	.	.	.
W1277	.	C	.	T	.	G	.	.	-	C	.	C	.	.	C	A	.	.	.	.	.	G	.	.
W1306	.	.	.	.	.	.	.	.	.	.	.	.	.	.	.	.	.	.	.	.	.	.	.	.
W1393	.	.	.	T	.	.	.	.	.	.	.	.	.	G	C	.	.	.	.	.	.	.	.	.
W1440	.	.	.	T	.	.	.	.	.	.	.	.	.	.	C	.	.	.	.	.	.	.	.	.
W1454	.	.	.	T	.	G	.	.	-	C	.	C	.	.	C	A	.	.	.	.	.	.	.	.
W1466	.	.	.	T	.	.	.	.	.	.	.	.	.	G	C	.	.	.	.	.	.	.	.	.
W1488	.	.	G	T	.	G	.	.	-	C	.	C	.	.	C	A	.	.	.	.	.	.	.	.
W1573	.	.	.	T	.	.	.	.	.	.	.	.	.	.	.	.	.	.	.	.	.	.	.	.
W1585	.	.	.	T	.	.	.	.	.	.	.	.	.	.	C	.	.	.	.	.	.	.	.	.
W1632	C	.	.	T	.	.	.	.	.	.	.	.	.	.	C	.	.	.	.	.	.	.	.	.
W1725	.	.	.	T	.	.	.	.	.	.	.	.	.	.	C	.	G	.	.	.	.	.	.	.

I:intron E:exon	I5	I5	I5	I5	I5	I5	I4	I3	I3	I2	I2	I2	I2	I2	I2	I2	I2	I2	I2	I2	I2	I2	I2	I2	I2	E2
Nucleotide position on BAC HN_550I12 contig3	44224	44263	44295	44306	44362	44363	44477	44674	44675	44787	44791	44798	44846	44853	44870	44879	44896-44897	44909	44912	44934	44950	44970	44978	45020	45025	45071
W_H602	A	G	C	C	C	T	A	G	A	A	C	G	A	G	T	A	TA	TA	A	G	T	A	G	G	T	G
W387	.	.	.	.	.	.	.	.	.	.	.	.	.	.	.	.	.	.	.	.	.	.	.	.	.	.
W395	.	.	.	.	.	.	.	.	G	.	T	.	T	T	C	.	.	--	G	.	.	.	.	.	.	.
WC394	.	.	.	.	.	.	.	.	.	.	.	.	.	.	.	.	.	.	.	.	.	.	.	.	.	.
W393	.	.	.	.	.	.	.	.	.	.	.	.	.	.	.	.	.	.	.	.	.	.	.	.	.	.
C064	G	C	.	.	.	.	.	.	.	.	.	A	.	.	C	.	.	.	.	.	.	.	.	C	.	A
C155	.	.	T	.	T	.	C	.	G	.	T	.	T	T	C	.	.	--	G	.	.	.	.	.	.	.
C211	.	.	T	.	T	.	C	.	G	.	T	.	T	T	C	.	.	--	G	.	.	.	.	.	.	.
C378	.	.	.	.	.	.	.	.	G	.	T	.	T	T	C	.	.	--	G	.	.	.	.	.	.	.
C351	.	.	.	.	.	A	.	.	G	.	T	.	T	T	C	.	.	--	G	.	.	.	.	.	C	.
W388	.	.	.	.	.	.	.	.	G	.	T	.	T	T	C	.	.	--	G	.	.	.	.	.	.	.
C075	.	.	.	.	.	.	.	.	G	.	.	.	.	.	C	.	.	.	.	.	.	.	.	.	.	.
W382	.	.	.	.	.	.	.	.	G	.	T	.	T	T	C	.	.	--	G	.	.	.	.	.	.	.
C153	.	.	.	.	.	.	.	.	G	.	T	.	T	T	C	.	.	--	G	.	.	.	.	.	.	.
C011	.	.	.	G	.	.	.	.	G	.	T	.	T	T	C	.	.	--	G	.	.	.	.	.	.	.
C025	.	.	.	G	.	.	.	.	G	.	T	.	T	T	C	.	.	--	G	.	.	G	.	.	.	.
C126	.	.	.	.	.	A	.	.	G	.	T	.	T	T	C	.	.	--	G	.	.	.	.	.	C	.
C021	.	.	.	.	.	A	.	.	G	.	T	.	T	T	C	.	.	--	G	.	.	.	.	.	C	.
W391	.	.	.	.	.	.	.	.	G	.	T	.	T	T	C	.	.	--	G	.	.	.	.	.	.	.
C030	.	.	.	G	.	.	.	.	G	.	T	.	T	T	C	.	.	--	G	.	.	G	.	.	.	.
C361	.	.	.	.	.	.	.	.	G	.	T	.	T	T	C	.	.	--	G	.	.	.	.	.	.	.
C353	.	.	.	.	.	.	.	.	G	.	T	.	T	T	C	.	.	--	G	.	.	.	.	.	.	.
C154	.	.	.	.	.	.	.	.	G	.	T	.	T	T	C	.	.	--	G	.	.	.	.	.	.	.
C302	.	.	.	.	.	.	.	.	G	.	T	.	T	T	C	.	.	--	G	.	.	.	.	.	.	.
WC398	.	.	.	.	.	.	.	.	G	.	T	.	T	T	C	.	.	--	G	.	.	.	.	.	.	.
C317	.	.	.	G	.	.	.	.	G	.	T	.	T	T	C	.	.	--	G	.	.	G	.	.	.	.
C350	.	.	T	.	T	.	C	.	G	.	T	.	T	T	C	.	.	--	G	.	.	.	.	.	.	.
C220	.	.	.	.	.	.	.	.	G	.	T	.	T	T	C	.	.	--	G	.	.	.	.	.	.	.
C_HN	.	.	.	.	.	.	.	.	G	.	T	.	T	T	C	.	.	--	G	.	.	.	.	.	.	.
C266	.	.	.	.	.	.	.	.	G	.	T	.	T	T	C	.	.	--	G	.	.	.	.	.	.	.
C043	.	.	.	.	.	.	.	.	G	.	T	.	T	T	C	.	.	--	G	.	.	.	.	.	.	.
C308	.	.	.	.	.	.	.	.	G	.	T	.	T	T	C	.	.	--	G	.	.	.	.	.	.	.
C325	.	.	.	.	.	.	.	.	G	.	T	.	T	T	C	.	.	--	G	.	.	.	.	.	.	.
W386	.	.	.	.	.	A	.	.	G	.	T	.	T	T	C	.	.	--	G	.	.	.	.	.	C	.
WC051	.	.	T	.	T	.	.	.	G	.	T	.	T	T	C	.	.	--	G	.	.	.	.	.	.	.
C172	.	.	T	.	T	.	.	.	G	.	T	.	T	T	C	.	.	--	G	.	.	.	.	.	.	.
C063	.	.	T	.	T	.	.	.	G	.	T	.	T	T	C	.	.	--	G	.	.	.	.	.	.	.
C125	.	.	T	.	T	.	.	.	G	.	T	.	T	T	C	.	.	--	G	.	.	.	.	.	.	.
C121	.	.	.	.	.	.	.	.	.	.	.	A	.	.	C	.	.	.	.	.	.	.	.	.	.	.
W0303	.	.	.	.	.	.	.	.	G	.	T	.	T	T	C	G	.	--	G	.	.	.	.	.	.	.
W0982	.	.	.	.	.	.	.	.	G	.	T	.	T	T	C	G	.	--	G	.	.	.	.	.	.	.
W1215	.	.	.	.	.	.	.	.	G	.	T	.	T	T	C	G	.	--	G	.	.	.	.	.	.	.
W1238	.	.	.	.	.	.	.	.	.	.	.	.	.	.	.	.	.	.	.	.	.	.	.	.	.	.
W1277	G	.	.	.	.	.	.	.	.	.	.	A	.	.	C	G	.	.	.	.	.	.	A	C	.	.
W1306	.	.	.	.	.	.	.	.	.	.	.	.	.	.	.	.	.	.	.	.	.	.	.	.	.	.
W1393	.	.	.	.	.	.	.	.	G	.	T	.	T	T	C	G	.	--	G	-	.	.	.	.	C	.
W1440	.	.	.	.	.	.	.	.	G	.	T	.	T	T	C	G	.	--	G	.	.	.	.	.	.	.
W1454	G	.	.	.	.	.	.	.	.	.	.	A	.	.	C	G	.	.	.	.	.	.	.	C	.	.
W1466	.	.	.	.	.	.	.	.	G	.	T	.	T	T	C	G	.	--	G	.	.	G	.	.	.	.
W1488	.	.	.	.	.	.	.	C	G	T	T	.	T	T	C	G	.	--	G	.	.	G	.	.	.	.
W1573	.	.	.	.	.	.	.	.	.	.	.	.	.	.	.	.	.	.	.	.	.	.	.	.	.	.
W1585	.	.	.	.	.	.	.	.	G	.	T	.	T	T	C	G	.	--	G	.	.	G	.	C	.	.
W1632	.	.	.	.	.	.	.	.	G	.	T	.	T	T	C	G	.	--	G	.	.	G	.	.	.	.
W1725	.	.	.	.	.	A	.	.	G	.	T	.	T	T	C	G	.	--	G	.	C	.	.	.	.	.

I:intron E:exon	I1	I1	I1	I1	I0	I0	I0	I0	I0	I0	I0	I0	I0	I0	I0	I0	I0	I0	I0	I0	I0	I0	I0	I0	I0	I0	I0	I0
Nucleotide position on BAC HN_550I12 contig3	45109	45125	45131	45167	45326	45355	45355-45357	45358-45384	45447	45449	45503	45508	45514	45539-45572	45575	45586	45633	45637	45639	45649	45651-45657	45658	45659-45678	45700	45701	45705	45719	45728
W_H602	A	A	A	-C--	G	GC	GC	TG	A	C	C	A	C	------	T	C	A	G	T	A	TGA	C	ATT	C	G	G	A	A
W387	.	.	.	.	.	.	.	.	.	.	.	.	.	.	.	.	.	.	.	.	.	.	.	.	.	.	.	.
W395	.	.	.	.	.	------	.	.	.	.	G	G	G	GCT	C	.	.	.	-	.	.	.	.	G	.	A	.	.
WC394	.	.	.	.	.	.	.	.	.	.	.	.	.	.	.	.	.	.	.	.	.	.	.	.	.	.	.	.
W393	.	.	.	.	.	.	.	.	.	.	.	.	.	.	.	.	.	.	.	.	.	.	.	.	.	.	.	.
C064	.	.	.	.	.	------	.	.	.	.	.	.	.	.	.	.	.	.	-	.	.	.	.	.	.	.	.	.
C155	G	.	.	.	.	------	.	.	.	.	.	.	.	.	.	.	.	.	-	.	.	G	.	G	.	A	C	C
C211	G	.	.	.	.	------	.	.	.	.	.	.	.	.	.	.	.	.	-	.	.	G	.	G	.	A	C	C
C378	.	.	.	.	.	------	.	.	.	.	.	G	.	GCT	C	.	.	.	-	.	.	G	.	.	.	.	.	.
C351	.	.	.	.	.	.	.	.	.	.	.	.	.	.	.	T	.	.	-	.	.	.	.	.	.	.	.	.
W388	.	.	.	.	.	.	.	.	.	.	G	G	G	GCT	C	.	.	.	-	.	.	G	.	G	.	A	C	C
C075	.	.	.	.	.	.	.	.	.	.	.	.	.	.	.	.	.	.	-	.	.	.	.	.	.	.	.	.
W382	.	.	.	.	.	.	.	.	.	.	G	G	G	GCT	C	.	.	.	-	.	.	G	.	G	.	A	C	C
C153	.	.	.	.	.	------	.	.	.	.	G	G	G	GCT	C	.	.	.	-	.	.	.	.	G	.	A	.	.
C011	.	.	.	.	.	------	.	.	.	.	G	G	G	GCT	C	.	.	.	-	.	.	.	.	G	.	A	.	.
C025	G	.	.	.	.	------	.	.	.	.	G	G	G	GCT	C	.	.	.	-	.	.	G	.	G	.	A	C	C
C126	.	.	.	.	.	.	.	.	.	.	.	.	.	.	.	.	.	.	-	.	.	.	.	.	.	.	.	.
C021	.	.	.	.	.	.	.	.	.	.	.	.	.	.	.	T	.	.	-	.	.	.	.	.	.	.	.	.
W391	G	.	.	CG	.	------	.	.	.	.	.	.	.	.	.	.	.	.	-	.	.	.	.	.	.	.	.	.
C030	G	.	.	.	.	------	.	.	.	.	G	G	G	GCT	C	.	.	.	-	.	.	G	.	G	.	A	C	C
C361	.	.	.	.	.	------	.	.	.	.	G	G	G	GCT	C	.	.	.	-	.	.	.	.	G	.	A	.	.
C353	.	.	.	.	.	------	.	.	.	.	G	G	G	GCT	C	.	.	.	-	.	.	.	.	G	.	A	.	.
C154	.	.	.	.	.	.	.	.	.	.	G	G	G	GCT	C	.	.	.	-	.	.	.	.	G	.	A	.	.
C302	.	.	.	.	.	------	.	.	.	.	G	G	G	GCT	C	.	.	.	-	.	.	.	.	G	.	A	.	.
WC398	.	.	.	.	.	------	.	.	.	.	G	G	G	GCT	C	.	.	.	-	.	.	.	.	G	.	A	.	.
C317	G	.	.	.	.	------	.	.	.	.	G	G	G	GCT	C	.	.	.	-	.	.	G	.	G	.	A	C	C
C350	G	.	.	.	.	------	.	.	.	.	.	.	.	.	.	.	.	.	-	.	.	G	.	G	.	A	C	C
C220	.	.	.	.	.	------	.	.	.	.	.	.	.	.	.	.	.	.	-	.	.	G	.	G	.	A	C	C
C_HN	.	.	.	.	.	------	.	.	.	.	G	G	G	GCT	C	.	.	.	-	.	.	.	.	G	.	A	.	.
C266	.	.	.	.	.	------	.	.	.	.	G	G	G	AGC	C	.	.	.	-	.	.	.	.	G	.	A	.	.
C043	.	.	.	.	.	------	.	.	.	.	G	G	G	GCT	C	.	.	.	-	.	.	.	.	G	.	A	.	.
C308	.	.	.	.	.	------	.	.	.	.	G	G	G	GCT	C	.	.	.	-	.	.	.	.	G	.	A	.	.
C325	.	.	.	.	.	------	.	.	.	.	G	G	G	GCT	C	.	.	.	-	.	.	.	.	G	.	A	.	.
W386	.	.	.	.	.	.	.	.	.	.	G	G	.	GCT	C	.	.	.	-	.	.	G	.	G	.	A	C	C
WC051	.	.	.	.	.	------	.	.	.	.	.	.	.	.	.	.	.	.	-	.	.	.	.	.	.	.	.	.
C172	.	.	.	.	.	------	.	.	.	.	.	.	.	.	.	.	.	.	-	.	.	G	.	G	.	A	C	C
C063	.	.	.	.	.	------	.	.	.	.	.	.	.	.	.	.	.	.	-	.	.	.	.	.	.	.	.	.
C125	.	.	.	.	.	------	.	.	.	.	.	.	.	.	.	.	.	.	-	.	.	.	.	.	.	.	.	.
C121	.	.	.	.	.	------	.	.	.	.	G	G	G	GCT	C	.	.	.	-	.	.	G	.	G	.	A	C	C
W0303	.	.	.	.	.	.	.	.	.	.	G	G	G	GCT	C	.	.	.	-	.	.	.	.	G	.	A	.	.
W0982	.	.	.	.	.	------	.	.	.	.	.	.	.	.	.	.	.	.	-	.	.	.	.	.	C	.	.	.
W1215	.	.	.	.	.	.	.	.	.	.	.	.	.	.	.	.	.	.	-	.	.	.	.	.	.	.	.	.
W1238	.	.	.	.	.	.	.	.	.	.	.	.	.	.	.	.	.	.	.	.	.	.	.	.	.	.	.	.
W1277	.	.	.	.	.	------	---	-----	C	A	.	.	.	.	.	.	.	.	-	.	.	G	.	G	.	A	C	C
W1306	.	.	.	.	.	.	.	.	.	.	.	.	.	.	.	.	.	.	.	.	.	.	.	.	.	.	.	.
W1393	.	.	.	.	.	.	.	.	.	.	G	G	.	GCT	C	.	.	.	-	.	.	G	.	G	.	A	C	C
W1440	.	.	.	.	.	.	.	.	.	.	.	.	.	.	.	.	.	.	-	.	.	.	.	.	.	.	.	.
W1454	.	.	.	.	.	.	.	.	.	.	.	.	.	.	.	.	.	.	-	.	.	.	.	.	.	.	.	.
W1466	.	.	.	.	.	.	.	.	.	.	G	G	G	GCT	C	.	.	.	-	T	.	G	.	G	.	A	C	C
W1488	G	.	.	.	-	.	.	.	.	.	.	.	.	.	.	.	.	.	-	.	.	.	.	.	.	.	.	.
W1573	.	.	.	.	.	------	.	.	.	.	G	G	G	GCT	C	.	G	.	-	.	.	G	.	G	.	A	C	C
W1585	.	.	.	.	.	------	.	.	.	.	G	G	G	GCT	C	.	.	.	-	.	.	G	.	G	.	A	C	C
W1632	G	T	.	.	.	.	.	.	.	.	G	G	G	GCT	C	.	.	.	-	.	.	G	.	G	.	A	C	C
W1725	G	.	.	-T--	.	------	.	.	.	.	G	G	G	GCT	C	.	.	.	-	.	-----	-	------	G	.	A	C	C

Supplementary Table 7| Information and annotation on amino acid sequences showing similarities to QSD1.

Abbreviation	Species	ID	Annotation	Position	Remark
Hv1	*Hordeum vulgare*	gi|326520203|dbj|BAK04026.1|	predicted protein	chromosome 5HL	QSD1
Hv2	*Hordeum vulgare*	gi|326525020|dbj|BAK07780.1|	predicted protein	chromosome 2HS	
Hv3	*Hordeum vulgare*	gi|1703227|sp|P52894.1|ALA2_HORVU	alanine aminotransferase	chromosome 1H?	AlaAT[24]
Hv4	*Hordeum vulgare*	gi|326491065|dbj|BAK05632.1|	predicted protein		
Hv5part	*Hordeum vulgare*	gi|326495904|dbj|BAJ90574.1|	predicted protein partial	chromosome 2H	
Os1	*Oryza sativa*	gi|115479309|ref|NP_001063248.1|		Os09g0433900	
Os2	*Oryza sativa*	gi|115481822|ref|NP_001064504.1|		Os10g0390500	
Os3	*Oryza sativa*	gi|297610424|ref|NP_001064505.2|		Os10g0390600	
Os4	*Oryza sativa*	gi|115473371|ref|NP_001060284.1|		Os07g0617800	
Os5	*Oryza sativa*	gi|115470235|ref|NP_001058716.1|		Os07g0108300	
Sb1	*Sorghum bicolor*	gi|242050886|ref|XP_002463187.1|	hypothetical protein	SORBIDRAFT_02g039340	
Sb2	*Sorghum bicolor*	gi|242039815|ref|XP_002467302.1|	hypothetical protein	SORBIDRAFT_01g023740	
Bd1	*Brachypodium distachyon*	gi|357158537|ref|XP_003578159.1|	PREDICTED: glutamate-glyoxylate aminotransferase 2-like		
Bd2	*Brachypodium distachyon*	gi|721610191|ref|XP_010235387.1|	PREDICTED: glutamate-glyoxylate aminotransferase 2-like isoform X2		
Bd3	*Brachypodium distachyon*	gi|357111762|ref|XP_003557680.1|	PREDICTED: glutamate-glyoxylate aminotransferase 2		
At1	*Arabipopsis thaliana*	gi|42562119|ref|NP_173173.3|	alanine aminotransferase		
At2	*Arabipopsis thaliana*	gi|14596229|gb|AAK68842.1|	alanine aminotransferase-like protein		
Mt1	*Medicago truncatula*	gi|357514319|ref|XP_003627448.1|:39-524	alanine aminotransferase		
Mt2	*Medicago truncatula*	gi|357485703|ref|XP_003613139.1|:9-475	glutamate-glyoxylate aminotransferase		

Supplementary Table 8| qPCR primers and conditions for expression analysis of *Qsd1*/*qsd1*.

Primer	Full length/partial cDNAs	Forward sequence (5'-3')	Reverse sequence (5'-3')	Anealing Temp (˚C)	Fragment size (bp)
Qsd1	AK372829	AGGACAAGATCGAGGCAATG	ATAATTTGCAGGGAGCGAAG	60	98
Qsd1/qsd1	AK372829	CTCTTTGCCCCAACACAAAT	GAATGCGTTCACCAAGGTCT	60	152
*HvEF-1α**	L11740.1	GCCAGATCAGCAACGGTTAT	ATCTTGACGAACCCAGCATC	60	120

* http://www.ncbi.nlm.nih.gov/pubmed/815979
